# Anti-Alzheimer Potential of a New (+)-Pinitol Glycoside Isolated from *Tamarindus indica* Pulp: In Vivo and In Silico Evaluations

**DOI:** 10.3390/metabo13060732

**Published:** 2023-06-07

**Authors:** Esraa M. Mohamed, Abeer H. Elmaidomy, Rania Alaaeldin, Faisal Alsenani, Faisal H. Altemani, Naseh A. Algehainy, Mohammad A Alanazi, Alaa Bagalagel, Abdulhamid Althagafi, Mahmoud A Elrehany, Usama Ramadan Abdelmohsen

**Affiliations:** 1 Department of Pharmacognosy, Faculty of Pharmacy, MUST, Giza 12566, Egypt; esraakadrymohamed@gmail.com; 2 Department of Pharmacognosy, Faculty of Pharmacy, Beni-Suef University, Beni-Suef 62511, Egypt; 3 Department of Biochemistry, Faculty of Pharmacy, Deraya University, University Zone, New Minia 61111, Egypt; rania.alaadin@deraya.edu.eg (R.A.); mahmoud.elrehany@deraya.edu.eg (M.A.E.); 4 Department of Pharmacognosy, College of Pharmacy, Umm Al-Qura University, Makkah 21955, Saudi Arabia; fssenani@uqu.edu.sa; 5 Department of Medical Laboratory Technology, Faculty of Applied Medical Sciences, University of Tabuk, Tabuk 71491, Saudi Arabia; faltemani@ut.edu.sa (F.H.A.); nalgehainy@ut.edu.sa (N.A.A.); m.alenezi@ut.edu.sa (M.A.A.); 6 Department of Pharmacy Practice, Faculty of Pharmacy, King Abdulaziz University, Jeddah 21589, Saudi Arabia; abagalagel@kau.edu.sa (A.B.); aalthagfi@kau.edu.sa (A.A.); 7 Department of Pharmacognosy, Faculty of Pharmacy, Deraya University, 7 Universities Zone, New Minia 61111, Egypt

**Keywords:** *Tamarindus*, tamarind, pinitol, Alzheimer

## Abstract

*Tamarindus indica* Linn (tamarind, F. Leguminosae) is one of the most widely consumed edible fruits in the world. Phytochemical investigation of tamarind pulp *n*-butanol fraction yielded one new (+)-pinitol glycoside compound **1** (25% *w*/*w*), and 1D, 2D NMR, and HRESIMS investigation were used to confirm the new compound’s structure. (+)-Pinitol glycoside showed anti-Alzheimer potential that was confirmed in prophylactic and treatment groups by decreasing time for the T-maze test; decreased TAO, brain and serum AChE, MDA, tau protein levels, and *β* amyloid peptide protein levels; and increasing GPX, SOD levels, and in vivo regression of the neurodegenerative features of Alzheimer’s dementia in an aluminum-intoxicated rat model. The reported molecular targets for human Alzheimer’s disease were then used in a network pharmacology investigation to examine their complex interactions and identify the key targets in the disease pathogenesis. An in silico-based analysis (molecular docking, binding free energy calculation (Δ*G*_Binding_), and molecular dynamics simulation) was performed to identify the potential targets for compound **1**. The findings of this study may lead to the development of dietary supplements for the treatment of Alzheimer’s disease.

## 1. Introduction

The majority of neurodegenerative disorders affecting the aged are amyloid-plaques, neurofibrillary tangles, cholinergic dysfunction, and oxidative stress. The most prevalent of these is Alzheimer disease (AD), which causes progressive neurocognitive deterioration and memory impairment (dementia) [1]. Patients experience a deterioration in their physical and cognitive abilities as they age, which may be related to a higher vulnerability to the cumulative effects of oxidative stress and inflammation [2]. Currently, only symptomatic treatments are available for AD. Three AChE inhibitors, donepezil, rivastigmine, galantamine, and memantine, are currently available and approved for the treatment of mild to moderate AD; however, they come with a number of side effects [3]. The “one change, one disease, one drug” paradigm is no longer appropriate because AD is a typical example of a complicated multifactorial disease [4]. There is a high demand for the discovery of novel natural products with the potential to protect against or even prevent this neurodegenerative disease, slow or even stop the disease’s progression and deterioration in its early stages, and/or lessen its side effects, all of which could promote healthy aging [5].

Given the critical functions of antioxidant chemicals in the treatment and prevention of illnesses connected to the oxidative stress that is produced by free radicals, research on plants with antioxidative potential has gained growing attention [6,7]. Antioxidants do, in fact, serve to scavenge free radicals that can interfere with cellular genetic material and destroy cellular membranes [6]. Natural products provide excellent chances to slow the progression and symptoms of AD [6]. The antioxidant, anti-inflammatory, anticholinesterase, and anti-amyloidogenic properties of plant-derived natural compounds such quercetin, berberine, epigallocatechin-3-gallate, huperzine A, resveratrol, and luteolin are of particular interest [8].

Inositols are naturally occurring cyclitols or polyols, and they can be found in the mammalian and plant kingdoms [9]. In terms of more specific chemical structure, these natural products are stereoisomers of hexahydroxy cyclohexane. The biological properties of inositols have been extensively studied, including insulin regulation, antidiabetic, antioxidant, antibacterial, female fertility enhancement, metabolic syndrome treatment, antidepressant, gastroprotective, hepatoprotective, hypolipidemic, and antiaging [10,11,12,13,14,15,16,17,18].

Tamarind is a native edible plant in Eastern Africa [19,20]. Recently, the positive anti-Alzheimer effects of metabolites (e.g., 4-phloroglucinol, 5-methoxybenzoicacid, 4-(3′-methoxyphloroglucinol), 5-hydroxybenzoic acid, along with 3,5-dihydroxyphenyl formate, 5-methoxy, 3-hydroxyphenyl formate, tartaric acid, gondoicacid, and *β*-sitosterol) isolated from tamarind pulp have been carefully established [7]. Moreover, from tamarind root bark, (+)-pinitol was previously isolated and characterized [21]. (+)-Pinitol is the inositol methyl ether derivative, which can be found in more than 20 plant sources, and its highest content is in carob pods, at 5.5% [22]. The biological properties of (+)-pinitol have also been extensively studied, including anti-Alzheimer, antiaging, antibacterial, anticancer, antidepressant, antidiabetic, antifibrotic, antihyperlipidemic, and anti-inflammatory activities [23,24,25,26,27,28,29,30,31,32].

By illustrating the mode of action using several in silico assays, the purpose of the current study was to emphasize the potential therapeutic and positive benefits of the new (+)-pinitol glycoside isolated from *Tamarindus indica* pulp. This was indicated by a regression in the neurodegenerative features of Alzheimer’s dementia in an Al-intoxicated rat model, together with further examination of tau protein and *β* amyloid peptide levels.

## 2. Materials and Methods

Plant materials and experiments were conducted in accordance with relevant institutional, national, and international guidelines.

### 2.1. Plant Material

We bought *T. indica* pulp at the market. Dr. Abd El-Halim A. Mohammed of the Horticultural Research Institute’s Department of Flora and Phytotaxonomy Research in Dokki, Cairo, Egypt, graciously recognized *T. indica*. At the Department of Pharmacognosy, Faculty of Pharmacy, Beni-Suef University, Egypt, a voucher specimen (2021-BuPD 77) was deposited.

### 2.2. Chemicals and Reagents

The solvents utilized in this study came from El-Nasr Company for Pharmaceuticals and Chemicals (Egypt) and included n-hexane (n-hex.), dichloromethane (DCM), ethyl acetate (EtOAC), n-butanol (n-but.), ethanol, and methanol (MeOH). Methanol-d4 (CD_3_OD-*d*_4_) and other deuterated solvents were bought from Sigma-Aldrich (Saint Louis, MO, USA) for spectroscopic studies. With the use of Sephadex LH-20 (0.25-0.1 mm, GE Healthcare, Sigma-Aldrich), column chromatography (CC) was carried out. Precoated silica gel 60 GF_254_ plates (E. Merck, Darmstadt, Germany; 20 × 20 cm, 0.25 mm in thickness) were used for thin-layer chromatography (TLC). Spraying the spots with para-anisaldehyde (PAA) reagent (85:5:10:0.5 absolute EtOH: sulfuric acid: glacial acetic acid: *para*-anisaldehyde), and then heating them to 110 °C, allowed the spots to be seen [33].

### 2.3. Spectral Analyses

At 400 and 100 MHz, respectively, proton ^1^H and ^13^C distortionless enhancement by polarization transfer-Q (DEPT-Q) NMR spectra were captured. Tetramethylsilane (TMS) was employed in methanol-*d*_4_ (CD_3_OD-*d*_4_) as an internal standard, with the residual solvent peak (*δ*_H_ = 3.34, 4.78; and *δ*_C_ = 49.9) serving as references. Bruker AG, Billerica, Massachusetts, USA, provided the Bruker Advance III 400 MHz, BBFO Smart Probe, and Bruker 400 MHz AEON Nitrogen-Free Magnet for the measurements. A DEPT-Q experiment was used to determine carbon multiplicities. A Shimadzu UV 2401PC spectrophotometer (Shimadzu Corporation—UV-2401PC/UV-2501PC, Kyoto, Japan) was used to measure the UV spectrum of methanol. An infrared spectrophotometer, model Jasco FTIR 300E, was used to measure the infrared (IR) spectra. An Acquity Ultra Performance liquid chromatography system connected to a Synapt G2 HDMS quadrupole time-of-flight hybrid mass spectrometer (Waters, Milford, MA, USA) was used to obtain HRESIMS data. 

### 2.4. Extraction and Fractionation of Tamarindus Indica Pulp

*Tamarindus indica* pulp (2 kg) was extracted using a rotary evaporator (Buchi Rotavapor R-300, Cole-Parmer, Vernon Hills, IL, USA) and five liters of 70% ethanol macerated at room temperature for three days each to yield one thousand grams of crude extract. The dry extract was successfully portioned with solvents of various polarities (n-Hex, DCM, EtOAC, and n-but) after being suspended in 700 mL of distilled water (H_2_O). Each step’s organic phase was individually evaporated under reduced pressure to produce the fractions I (10.0 g), II (7.0 g), III (16.0 g), and IV (500.0 g), respectively. The leftover mother liquor was then concentrated to produce the aqueous fraction (V). The final fractions were all stored at 4 °C for biological and phytochemical analysis [7,34,35,36,37,38].

### 2.5. Isolation and Purification of Compounds

Fraction IV (50 g) was further purified on a Sephadex LH_20_ column (0.25–0.1 mm, 400 × 0.5 cm, 400 gm), which was eluted with MeOH to afford compound **1** (49.5 g) (content in the pulp, at 25.0% *w*/*w*).

3-*O*-[[3′-*O*-[*β*-d-glucopyranosyl-(1″-3′)]-[6′-*O*-[*β*-d-fructofuranosyl–(1‴-6′)]-*α*-d-glucopyranosyl]-(+)-pinitol (1): yellow powder; [UV (MeOH) *λ*_max_ (log_ε_) 270 (6.0), 300 (6.5) nm; IR υ_max_ (KBr) 3429, 3100, 3030, 1680, 1600 cm^−1^; NMR data; see Table 1; HRESIMS *m*/*z* 681.2454 [M + H]^+^ (calc. for C_25_H_45_O_21_, 681.2453).

### 2.6. Acid Hydrolysis and Sugar Analysis

Hydrolysis of sugars and GC-MS analysis of derivatives was performed according to Abbet et al. (2011) [39]. Compound **1** (1.0 mg) was hydrolyzed. After heating at 100 °C for 1 h in 2 M TFA (1 mL), the mixture was extracted with CH_2_Cl_2_ (3 × 1.0 mL). The aq. phase was freeze-dried, and redissolved in dry pyridine (200 mL) containing 5 mg/mL L cysteine methyl ester hydrochloride. The reaction mixture was heated at 60 °C for 1 h, followed by silylation with hexamethyldisilazane and chlorotrimethylsilane (Fluka) in pyridine (3:1:10, 300 mL) at 60 °C for 30 min. [40]. After silylation, pyridine was evaporated, and the solid residue extracted with *n*-hexane. GC-MS analysis was performed on a 5890 Series II gas chromatograph coupled to a HP 5971A mass detector (Hewlett Packard, Palo Alto, CA, USA). The separation was carried out on a DB-225 MS column (30 m × 0.25 mm, I.D., Waters, Taunton, MA, USA); column temp. 150 °C for 2 min, and then a gradient of 58 °C/min to 210 °C, then 10 °C/min to 240 °C. Comparison of the retention times of derivatized reference sugars with those obtained from samples resulted in (+)-pinitol (Rt 23.48 min), D-glucose (Rt 28.64 min), and D-fucose (Rt 25.96 min) in the tested compound.

### 2.7. Animals and Ethics

The Laboratory Animal Centre at Deraya University provided 32 adult male Wistar rats (12–15), weighing between 150 and 200 g. The Experimental Animal Centre and Research Ethics Committee, Deraya University, Minia, Egypt (12/2022 approved on 1 August 2022) developed the standards for animal care and study protocols. All rats were kept in groups of eight and kept on a 12-h light/dark cycle in an animal room with temperature and pressure controls.

### 2.8. Experimental Design

The animals were divided into four groups of eight rats each, and they received the following oral treatments for 21 days: Group (1): Normal healthy rats served as negative control; Group (2): Alzheimer disease (AD)-induced rats received AlCl_3_ orally at a dose of 17 mg/kg body weight daily, as described before [41]; Group (3): AD-induced rats received (+)-pinitol glycoside orally (100 mg/kg) from day 1 as prophylactic approach [42]; Group (4): AD-induced rats followed by (+)-pinitol glycoside treatment orally (100 mg/kg) for another 21 days.

Blood samples were taken at the conclusion of the experiment right before the rats were sacrificed for additional biochemical testing. Additionally, the entire brain was quickly separated into two parts and dissected on a glass dish that had been chilled with ice. For subsequent Western blotting examination, the first part was maintained at 80 °C. In phosphate-buffered saline pH (7.00), the second portion was homogenized using a Branson Digital Sonifier SFX 550 (EMERSON, Ferguson, MO, USA). To prepare the homogenate’s clear supernatant for acetyl choline esterase measurement, the homogenate was centrifuged at 4000 RPM for 40 min at 4 °C.

### 2.9. T-Maze Test

The T-maze test was utilized to evaluate the neurocognitive function of rats according to Deacon and Rawlins [43]. Before setting up this experiment, animals were not provided with food for 24 h with only water to drink. All animals were subjected to the T-maze test. The experiment was done thrice; at zero time before the induction of AlCl_3_, after 24 h of the first dose of ALCl_3_, and at the end of the experiment. Behavioral observations were recorded before and at the end of the experiment.

### 2.10. Biochemical Analysis

According to the manufacturer’s instructions, serum total antioxidant activity (TAO) was measured using a total antioxidant colorimetric test kit (#E-BC-K801-M, Elabscience, Houston, TX, USA). According to manufacturer’s instructions, the acetyl choline esterase activity kit (#E-BC-K174-M, Elabscience, Houston, TX, USA) was used to measure the activity of the AchE enzyme in the brain and serum.

### 2.11. ELISA Assays

Serum GPX, SOD, and MDA were determined according to the kits manufacturer’s instructions (#MBS744364, MyBioSource, San Diego, CA, USA), (#MBS036924, MyBioSource, San Diego, CA, USA), and (#MBS268427, MyBioSource, San Diego, CA, USA), respectively. Protein tissue homogenates were used for evaluating the protein levels of phosphorylated and total tau and *β* amyloid peptide utilizing rat tau protein ELISA kit (#MBS029585, MyBioSource, San Diego, CA, USA), (#MBS725098, MyBioSource, San Diego, CA, USA), and (#MBS726579, MyBioSource, San Diego, CA, USA).

### 2.12. In Silico Investigation

#### 2.12.1. Prediction of the Potential Targets

The isomeric structure of compound **1** was prepared by ChemDraw [44] and then submitted to the Swiss Targetto Pharm Mapper (http://www.lilab-ecust.cn/pharmmapper/, accessed on 20 March 2022) [45] to obtain the potential targets in the organism “*Homo sapiens*” (Table S1).

#### 2.12.2. Possible Targets of Alzheimer’s Disease

AD’s target proteins (Table S1) were collected from the following four databases: Gene Cards (https://www.genecards.org/, accessed on 20 March 2022) [46]; Therapeutic Target Database (TTD, http://db.idrblab.net/ttd/, accessed on 20 March 2022) [47]; Comparative Toxicogenomics Database (CTD, http://ctdbase.org/, accessed on 20 March 2022 ); and the Drug Bank database (https://www.drugbank.ca/, accessed on 20 March 2022) [48]. The word “Alzheimer Disease” was selected as the keyword, and the species was limited as “*Homo sapiens*”. The targets that repeated at least two times were selected.

#### 2.12.3. Molecular Docking MD Simulation and Network Construction

Docking was carried out using AutoDock Vina software, and MD simulations were performed using Desmond software, while the construction of PPI network was carried out using Cytoscape. The detailed descriptions of these procedures can be found in the Supplementary Materials [49,50,51,52,53,54,55,56,57].

### 2.13. Statistical Analysis

Standard deviations (SD) were used to report the mean for all data sets. Utilizing Co-State for Windows version 8 and one-way ANOVA software, the data were statistically checked for normal distribution. The values of different letters are statistically significant at *p* <0.001.

## 3. Results

### 3.1. Phytochemical Investigation 

**Figure 1:**
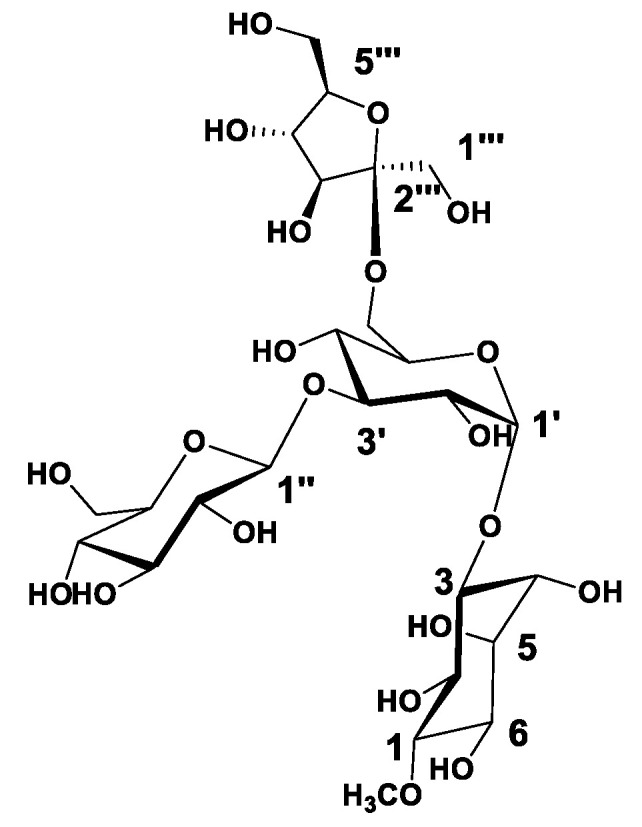
Structures of the compound **1** isolated from *Tamarindus indica* pulp.

**Figure 2:**
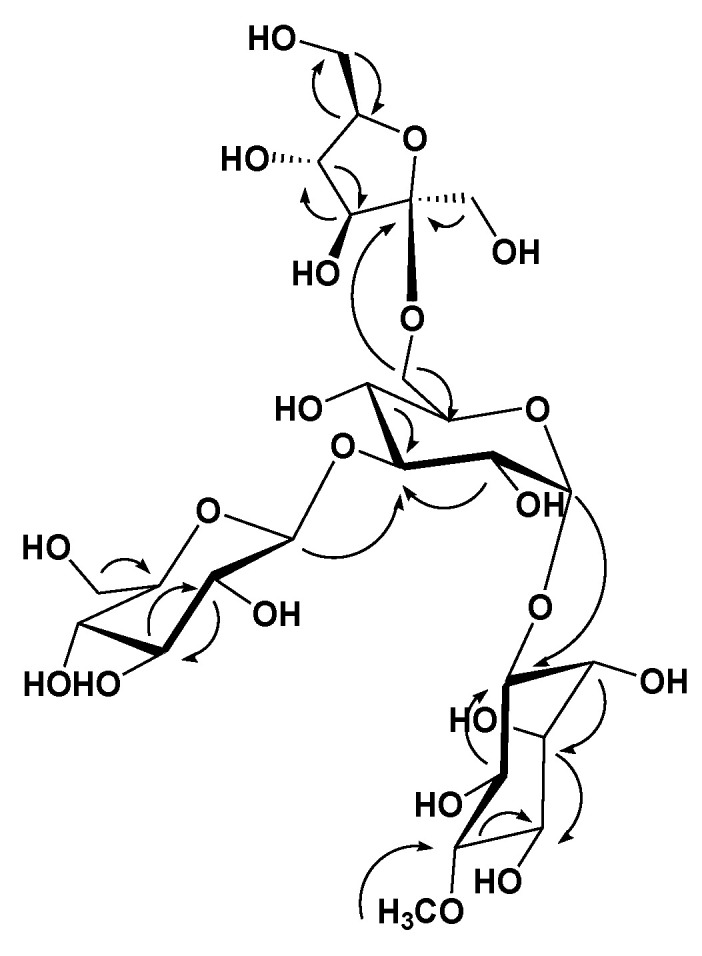
Selected HMBC (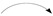) correlations of compound **1**.

Analysis of the HRESIMS, 1D, and 2D NMR data for compound **1** suggested possible oligosaccharide core scaffold [58]. The HRESIMS data for compound **1** showed an adduct pseudo-molecular ion peak at *m*/*z* 681.2454 [M + H]^+^, (calc. for C_25_H_45_O_21_, 681.2453), suggesting four degrees of unsaturation. The ^1^H and DEPT-Q ^13^C NMR data (Table 1, Figures S1 and S2) along with the Heteronuclear Single Quantum Correlation Experiment (HSQC) data (Figure S3) suggested seven characteristic resonances appeared as six oxymethine groups at *δ*_H_ 3.28 (1H, overlapped) *δ*_C_ 84.4, *δ*_H_ 3.92 (1H, overlapped) *δ*_C_ 73.0, *δ*_H_ 4.55 (1H, m) *δ*_C_ 73.3, *δ*_H_ 3.71 (1H, m) *δ*_C_ 72.2, *δ*_H_ 3.70 (1H, overlapped) *δ*_C_ 74.5, *δ*_H_ 3.81 (1H, overlapped) *δ*_C_ 71.5, and one methoxy group appeared at *δ*_H_ 3.63 (1H, s) *δ*_C_ 60.6, suggesting the characteristic core structure for a cyclitol, a cyclic polyol, (+)-pinitol unit that was previously isolated from *T. indica* bark [21]. NMR data also showed five oxymethine groups at *δ*_H_ 5.14 (1H, d, *J* = 3.5) *δ*_C_ 93.5, *δ*_H_ 3.81 (1H, overlapped) *δ*_C_ 71.4, *δ*_H_ 4.06 (1H, overlapped) *δ*_C_ 77.2, *δ*_H_ 3.81 (1H, overlapped) *δ*_C_ 69.1, *δ*_H_ 3.81 (1H, overlapped) *δ*_C_ 71.6, and one oxymethylene group at *δ*_H_ 3.62, 4.04 (2H, m) *δ*_C_ 64.0, suggesting the characteristic core structure for *α*-d-glucopyranosyl unit [19,59,60,61]. NMR data also showed five oxymethine groups at *δ*_H_ 4.51 (1H, d, *J* = 8) *δ*_C_ 97.7, *δ*_H_ 3.62 (1H, overlapped) *δ*_C_ 73.8, *δ*_H_ 3.16 (1H, t) *δ*_C_ 76.3, *δ*_H_ 3.87 (1H, overlapped) *δ*_C_ 70.8, *δ*_H_ 3.35 (1H, overlapped) *δ*_C_ 77.5, and one oxymethylene group at *δ*_H_ 3.81, 4.04 (2H, m) *δ*_C_ 62.4, suggesting the characteristic core structure for *β*-d-glucopyranosyl unit [60,62]. NMR data also showed three oxymethine groups at *δ*_H_ 3.35 (1H, overlapped) *δ*_C_ 77.6, *δ*_H_ 3.31 (1H, m) *δ*_C_ 71.2, *δ*_H_ 3.78 (1H, overlapped) *δ*_C_ 82.7, two oxymethylene groups at *δ*_H_ 3.50, 3.69 (2H, m) *δ*_C_ 65.5, *δ*_H_ 3.51, 4.04 (2H, m) *δ*_C_ 64.3, and one quaternary carbon appeared at *δ*_C_ 99.0, suggesting the characteristic core structure for *β*-d-fructofuranosyl unit [63]. The Heteronuclear Multiple Bond Correlation (HMBC) experiment (Figure S4) showed the ^3^*J*-HMBC correlation of the proton H-1′ *δ*_H_ 5.14 (*δ*_C_ 93.5) with CH-3 (*δ*_C_ 73.3), confirming the connections of the (+)-pinitol moiety at C-1′ of the *α*-d-glucopyranosyl moiety (Figure 1 and Figure 2). Additionally, HMBC showed ^3^*J*-HMBC correlation of the proton H-1″ *δ*_H_ 4.51 (*δ*_C_ 97.7) with CH-3′ (*δ*_C_ 77.2), confirming the connections of *β*-d-glucopyranosyl moiety at C-3′ of the *α*-d-glucopyranosyl moiety. Moreover, HMBC, showed ^3^*J*-HMBC correlation of the proton H-6′ *δ*_H_ 3.62, 4.04 (*δ*_C_ 64.0) with CH-2‴ (*δ*_C_ 99.0), confirming the connections of *β*-d-fructofuranosyl moiety at C-6′ of the *α*-d-glucopyranosyl moiety. Accordingly, compound **1** identified as 3-*O*-[[3′-*O*-[*β*-d-glucopyranosyl-(1″-3′)]-[6′-*O*-[*β*-d-fructofuranosyl–(1‴-6′)]-*α*-d-glucopyranosyl]-(+)-pinitol (Figure 1 and Figure 2).

### 3.2. Behavioral Assessment Using the T-Maze Test

**Figure 3:**
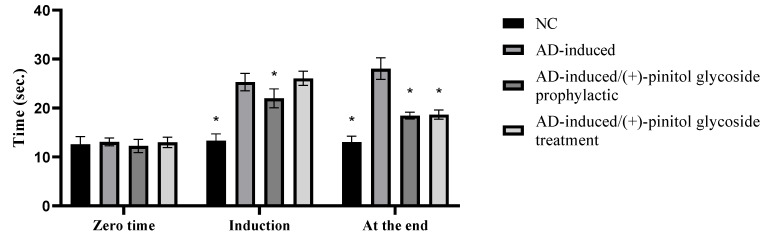
Prophylactic and therapeutic effect of (+)-pinitol glycoside on time spent in T-maze by different animals. Data represent mean ± SD (*n* = 8). Significant difference was analyzed by the one-way ANOVA test followed by the post hoc Dunnett test, where * indicates *p* < 0.001, compared to the AD-induced group.

As shown in Figure 3, our findings showed a significant (*p* < 0.001) increase in time (s) taken by animals to reach food during the induction stage in the AD-induced group, 25.31 ± 1.78 s, compared to the NC group, while the prophylactic ingestion of (+)-pinitol glycoside showed a notable (*p* < 0.001) decrease in time for rats to reach their food during the induction stage, 21.98 ± 1.92 s, compared to the AD-induced group. Interestingly, at the end of the experiment, the AD-induced group showed significant elevation in time to 28.06 ± 2.22 s compared to the NC group, while the (+)-pinitol glycoside-prophylactic and (+)-pinitol glycoside-treated groups showed a notable (*p* < 0.001) decrease in time, 18.45 ± 0.68 s and 18.66 ± 0.94 s, respectively, compared to the AD-induced group.

### 3.3. Biochemical Analysis

**Figure 4:**
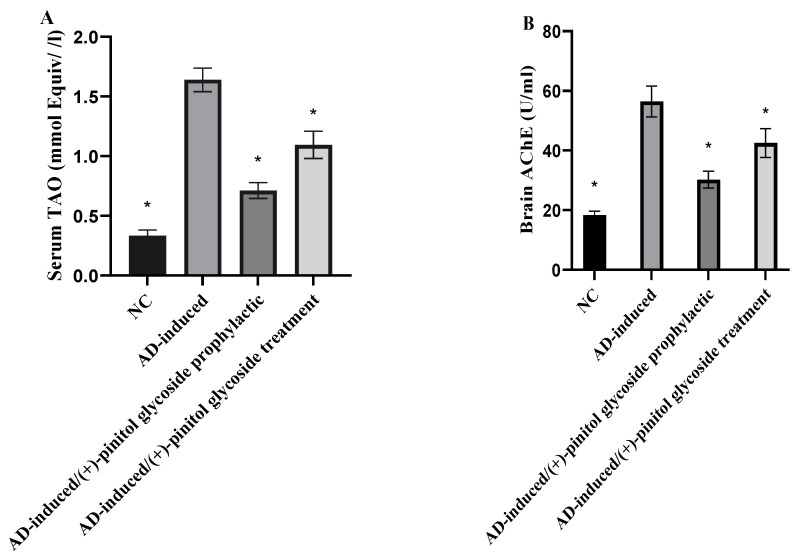

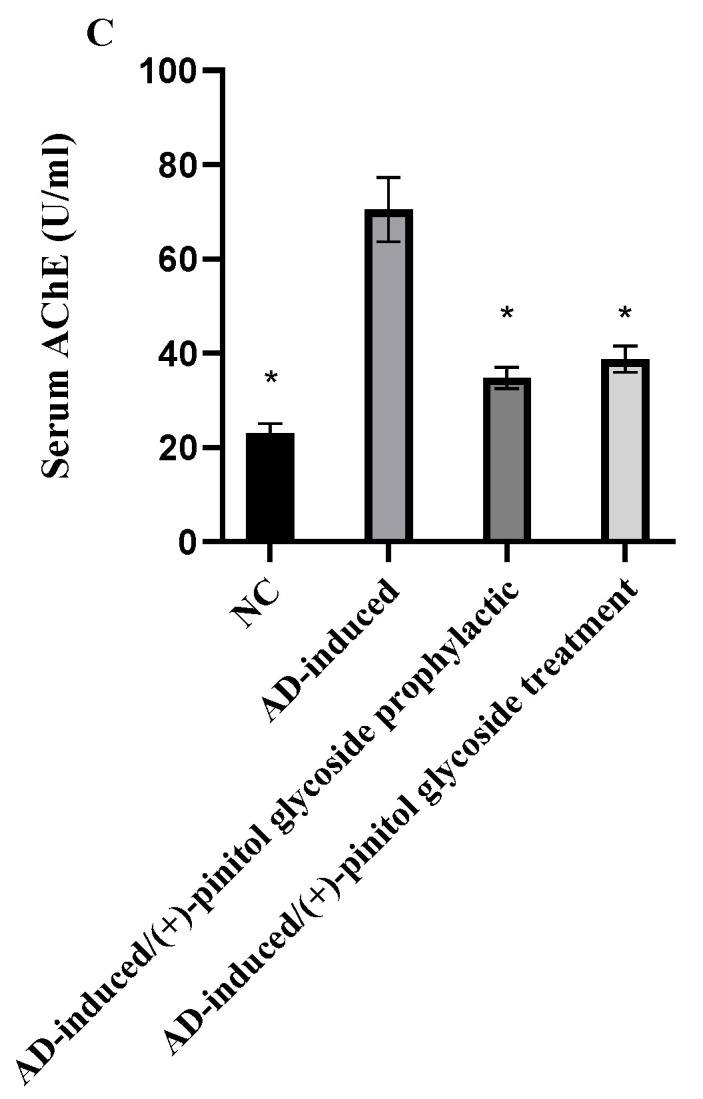
Prophylactic and therapeutic effect of (+)-pinitol glycoside on serum TAO (**A**), serum AChE activity (**B**), and brain AChE (**C**) activity in the different groups. Data represent mean ± SD (*n* = 8). Significant difference was analyzed by the one-way ANOVA test followed by the post hoc Dunnett test, where * indicates *p* < 0.001, compared to the AD-induced group.

TAO, serum AChE, and brain AChE levels were evaluated in the different groups to examine the prophylactic and treatment activity of (+)-pinitol glycoside on the AD-induced groups. As shown in Figure 4, TAO, brain AChE, and serum AChE levels were significantly (*p* < 0.001) elevated in the AD-induced groups to 1.64 ± 0.09 mmol Equiv/l, 56.41 ± 5.21 U/mL, and 70.42 ± 6.83 U/mL, respectively, compared to the NC group. Prophylactic ingestion of (+)-pinitol glycoside lowered (*p* < 0.001) serum TAO, brain AChE, and serum AChE to 0.71 ± 0.06 mmol Equiv/l, 30.21 ± 0.8 U/mL, and 34.76 ± 2.31 U/mL, respectively, compared to the AD-induced group. Additionally, treatment with (+)-pinitol glycoside showed notable (*p* < 0.001) inhibition to 1.09 ± 0.11 mmol Equiv/l, 42.52 ± 4.87 U/mL, and 38.74 ± 2.81 U/mL for TAO, brain AChE, and serum AChE, respectively.

### 3.4. Evaluation of Oxidative Markers

**Figure 5:**
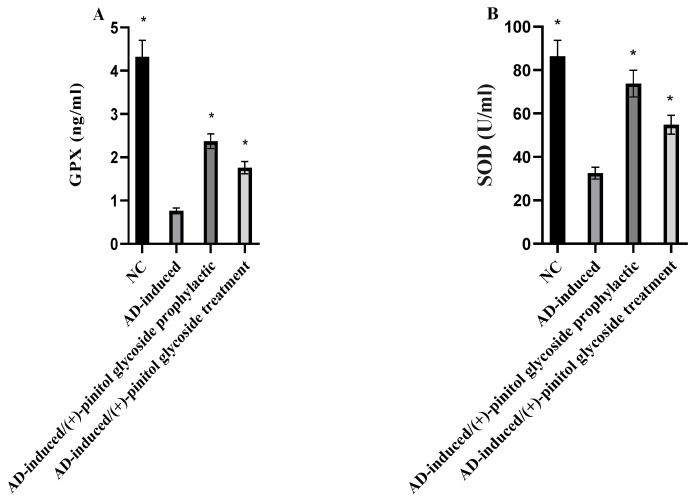

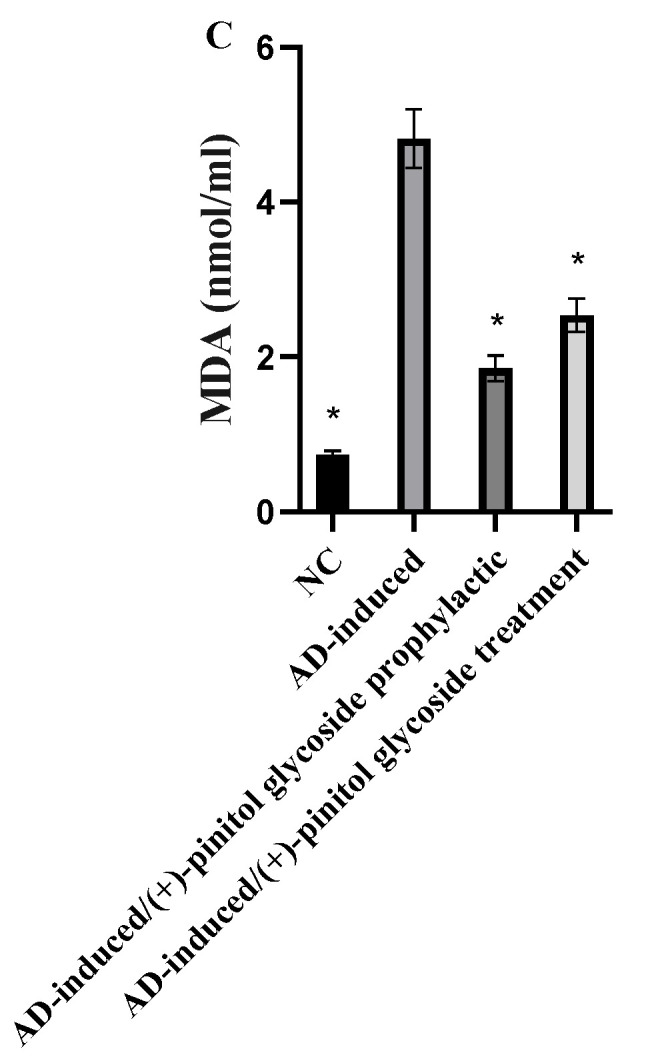
Prophylactic and therapeutic effect of (+)-pinitol glycoside on serum GPX (**A**), SOD (**B**), and MDA (**C**) in the different groups. Data represent mean ± SD (*n* = 8). Significant difference was analyzed by the one-way ANOVA test followed by the post hoc Dunnett test, where * indicates *p* < 0.001, compared to the AD-induced-group.

Serum GPX, SOD, and MDA levels were examined to evaluate the oxidative stress status during prophylaxis and treatment with (+)-pinitol glycoside in AD-induced rats. As shown in Figure 5A, B, GPX and SOD were notably (*p* < 0.001) decreased in the AD-induced group, compared to the NC group. During (+)-pinitol glycoside prophylaxis and treatment, serum levels of GPX and SOD were significantly (*p* < 0.001) elevated, compared to the AD-induced group. Regarding MDA levels, the AD-induced group showed a notable (*p* < 0.001) increase in their serum levels, compared to the NC group. However, the prophylactic and treatment ingestion of (+)-pinitol glycoside showed inhibition (*p* < 0.001) in MDA levels.

### 3.5. Evaluation of Tau and Amyloid Peptide

**Figure 6:**
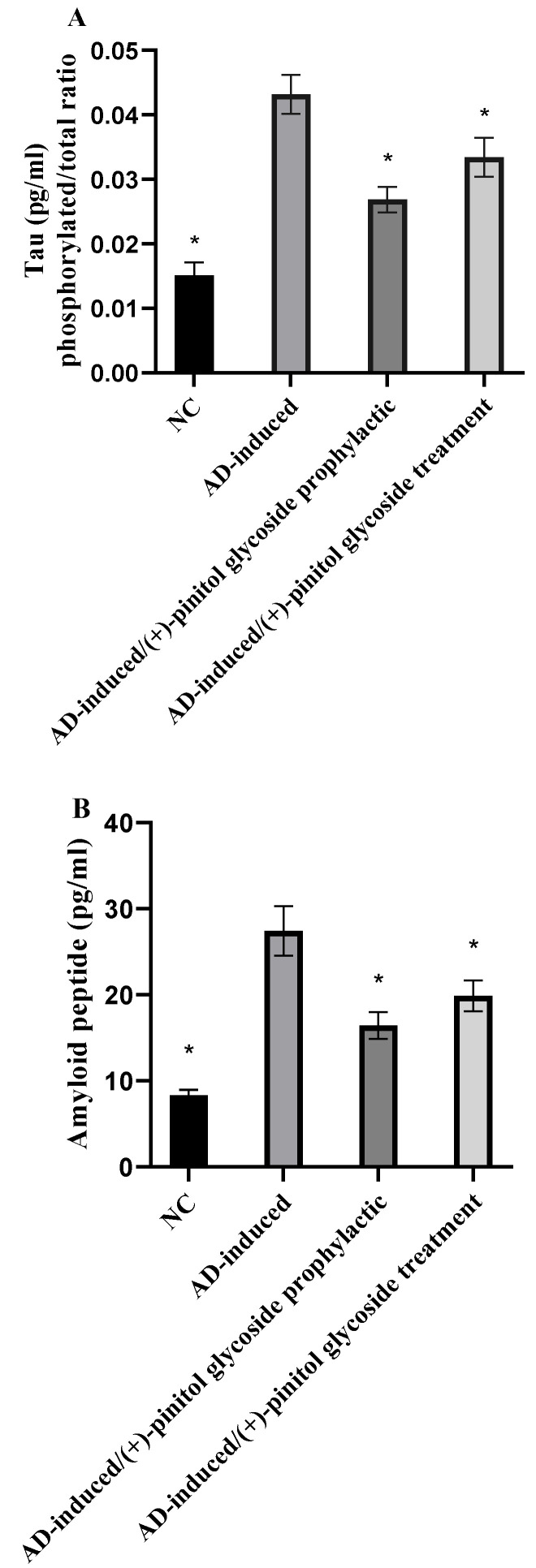
Brain tissue levels of tau (phosphorylated/total) (**A**) and *β* amyloid peptide proteins (**B**). Data represent mean ± SD (*n* = 8). Significant difference was analyzed by the one-way ANOVA test followed by the post hoc Dunnett test, where * indicates *p* < 0.001, compared to the AD-induced group.

Tau protein and *β* amyloid peptide were evaluated in the present study to examine the prophylactic and treatment activity of (+)-pinitol glycoside on the AD-induced groups. As shown in Figure 6, tau protein level was calculated as the ratio of phosphorylated to total tau. Tau and *β* amyloid peptide protein levels were notably (*p* < 0.001) elevated in the AD-induced groups, while showing a significant (*p* < 0.001) decrease in the (+)-pinitol glycoside-prophylactic and treated groups.

### 3.6. In Silico-Based Study

#### 3.6.1. PPI Network of the Predicted Targets and KEGG-Based Enrichment Analysis

To identify all human-based proteins associated with Alzheimer’s disease, we searched for them in the Toxicogenomics (https://ctdbase.org/, accessed on 20 March 2022) and the GeneCards databases (https://www.genecards.org/, accessed on 20 March 2022) in addition to the previously published literature. We used the Cytoscape software to construct a protein–protein interaction (PPI) network among the retrieved 83 proteins found in the literature and the databases to have direct links to human Alzheimer’s disease (Table S1).

**Figure 7:**
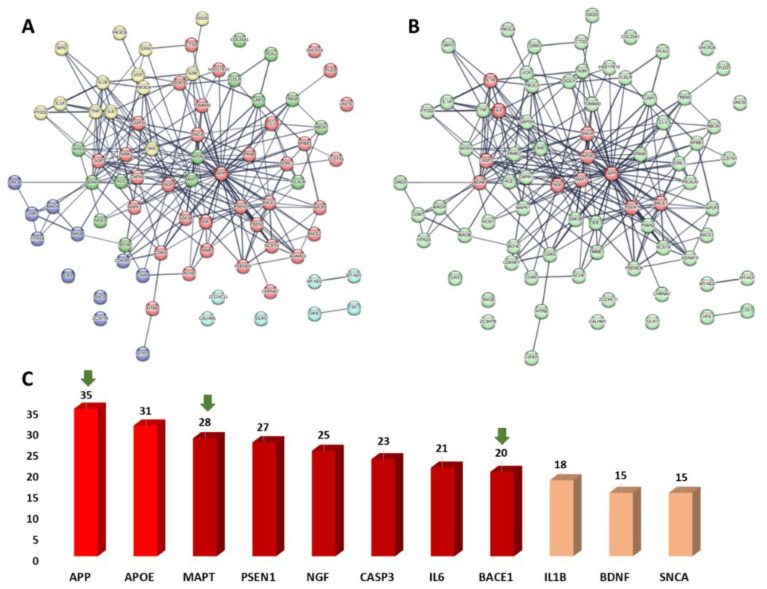
(**A**) Human Alzheimer’s disease PPI network. This network consists of 80 nodes and 256 edges with an average node degree of 6.4. In this network, the 83 Alzheimer-related proteins collected were clustered into five clusters according to their signaling pathways: blue nodes are for the enzymatic degradation of dopamine by monoamine oxidase and by COMT; green nodes are involved in the signaling by receptor tyrosine kinases; red nodes are for proteins involved in the amyloid fiber formation; yellow nodes are involved in the interleukin-10 signaling; cyan nodes are involved in the degradation of the extracellular matrix. (**B**) Human Alzheimer’s disease PPI network showing the top-interacting nodes (13.25% of all interacting nodes, i.e., hub proteins, red nodes). (**C**): The top 13.25% interacting-nodes (i.e., hub nodes arranged by their degree value). Green arrows represent the proteins predicted as probable targets for the anti-Alzheimer natural products investigated in the presented study. The thickness of the lines (i.e., edges) represents the degree of confidence (i.e., the strength of data support).

In Figure 7, we can see that the generated PPI network had many connections, with 256 edges connecting 80 nodes and an average node degree of 6.4 and a local clustering coefficient of 0.42. Proteins and/or genes with high degrees of interaction are usually the most important and relevant molecular targets (i.e., hub proteins or genes) in a particular network, and hence, targeting such proteins in Alzheimer’s disease might improve the likelihood for developing successful therapeutic strategies. As a result, we highlighted the top 13% (11 proteins) of the most heavily interacted molecular targets (i.e., hub proteins) ranked by their degree value (Figure 7B,C).

Additionally, we classified the proteins in the present network according to their involvement in the different signaling pathways related to the disease. This protein enrichment analysis was carried out according to the KEGG database (https://www.genome.jp/kegg/pathway.html, accessed on 20 March 2022). As shown in Figure 7A, the retrieved Alzheimer’s disease-related proteins were clustered into five groups according to their signaling pathways involved in the pathogenesis and/or the pathophysiology of the disease: (i) the enzymatic degradation of dopamine by monoamine oxidase and by COMT; (ii) the signaling mediated by receptor tyrosine kinases; (iii) the amyloid fiber formation; (iv) the interleukin-10 signaling pathway; and (v) the degradation of the extracellular matrix.

Taken together, the present Alzheimer’s disease PPI network provided a brief outline of the interacting proteins and the signaling pathways associated with them, indicating the key proteins that can be considered critical to the disease development, and hence, good targets for future drug development.

#### 3.6.2. Prediction of AD Target Proteins

In order to outline its anti-Alzheimer potential, compound **1** was subjected to a number of in silico-based experiments. First, we suggested a number of targets (82 targets, Table S1) relevant to Alzheimer disease using Gene Cards and KEGG [64]. Subsequently, the modeled structure of compound **1** was submitted to PharmMapper Prediction software to reveal its probable correlation with these suggested targets [65]. We set a fit score of 1 as the cutoff for the protein to be considered as a probable target for compound **1**.

From the 82 protein targets that were suggested to be highly relevant to AD, 11 proteins were predicted as potential targets for compound **1** (Table S2). Three of these predicted targets (i.e., APP, BACE1, and MAPT) were found to be highly interacting proteins (i.e., hub proteins) in the constructed Alzheimer’s disease PPI network (Figure 7B,C).

To refine these pharmacophore-based preliminary virtual screenings, we carried out molecular docking and MD simulation experiments for these predicted targets in association with compound **1**. Proteins that received docking scores <−7 kcal/mol with compound **1,** and their calculated absolute binding free energies (Δ*G*_binding_) were also <−7 kcal/mol, were considered as targets for compound **1**. Accordingly, only *β*-secretase (BACE1) and acetylcholine esterase (ACHE) were considered as potential targets for compound **1** (Table S2).

#### 3.6.3. Analysis of Possible Molecular Mechanisms

**Figure 8:**
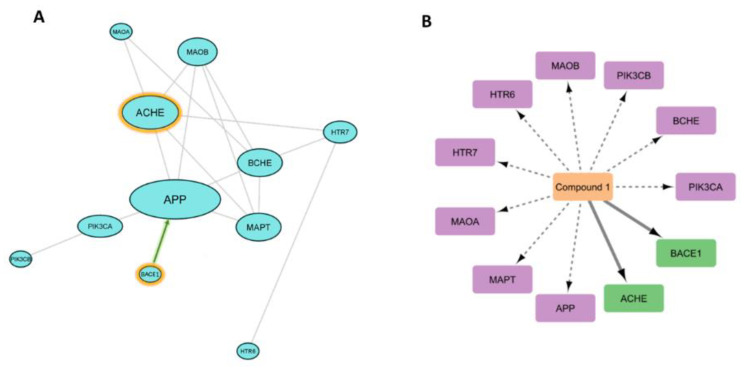
(**A**) PPI network (*p* < 8 × 10^−16^). Network nodes represent predicted protein targets, and the edges represent protein–protein interactions. The size of nodes represents the connectivity of each protein: the larger the node size, the greater its connectivity to other nodes. Orange-edged nodes represent the protein targets that received the highest scores in terms of their affinity in binding with compound **1**. The green bold arrow between BACE1 and APP indicates that BACE1 is the main catalytic enzyme responsible for the production of APP. (**B**) CPI network represents the interactions between compound **1** and its predicted protein targets by PharmMapper (i.e., dotted edges). Thick solid bold edges represent the interactions (i.e., dockings) with targets (green nodes) that were validated by MDS-based experiments.

In order to investigate how these predicted targets interact with each other and what is (are) the key target(s) in AD pathogenesis, we constructed a sub-protein–protein interactions (PPI) network between these predicted targets (Table S2, Figure 8). The sub-PPI analysis revealed that *β*-amyloid (APP) and acetylcholine esterase (ACHE) showed the most connections among the other 11 proteins, and this finding is highly consistent with the many reports that have described cholinergic activity and *β*-amyloid aggregation as a hallmark in AD pathogenesis [66]. *β*-secretase (BACE1) is the key hydrolytic enzyme responsible for the formation of APP [67], and hence, inhibition of such enzyme can lead eventually to suppress the most important protein in our PPI analysis (Figure 8).

#### 3.6.4. Binding Mode Analysis

**Figure 9:**
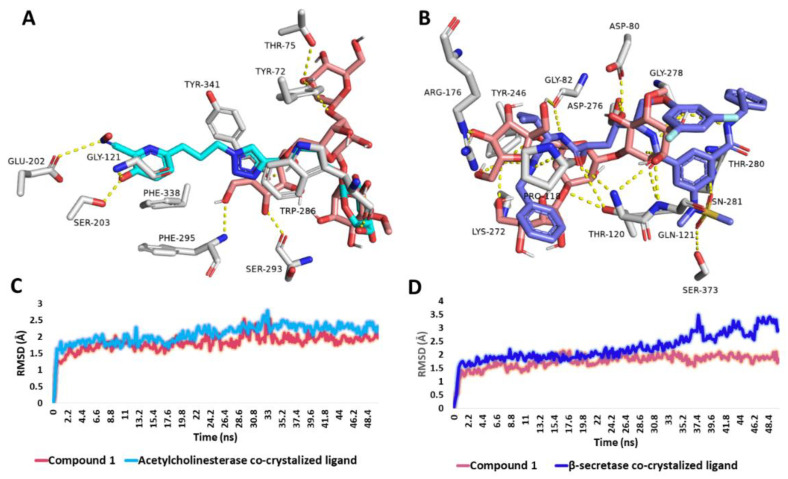
(**A**,**B**) Binding modes of compound **1** (brick red-colored structure) inside the active sites of acetylcholine esterase (PDB ID: 1qti) and *β*-secretase (PDB ID: 3ixj) in alignment with the co-crystallized inhibitor of each enzyme (cyan and blue structures, respectively). (**C**,**D**) RMSDs of compound **1** in comparison with the co-crystallized inhibitors inside the active sites of acetylcholine esterase and *β*-secretase during the course of the 50 ns-long MD simulation.

Further looking into the binding modes of compound **1** inside the active sites of *β*-secretase (PDB ID: 3ixj) [68] and acetylcholine esterase (PDB ID: 1qti) [69] revealed that the structure of compound **1** was able to achieve molecular interactions comparable with that of the co-crystallized ligands (Figure 9). In regard to acetylcholine esterase, compound **1** shared two H-bonds with the co-crystallized ligand, i.e., TRP-286 and TYR-341. In addition, it formed four extra H-bonds with SER-293, PHE-295, TYR-72, and THR-75 (Figure 9A). Fifty nanosecond-long MD simulations revealed that both compound **1** and the co-crystallized ligand were able to establish stable bindings inside the enzyme’s active site with low fluctuations and an average RMSD of ~1.6 Å (Figure 9C). 

On the other hand, compound **1** was highly interacted inside *β*-secretase’s active site through H-bonding, where it was partially aligned with the enzyme’s co-crystallized ligand (Figure 5B). Compound 1 shared six H-bonding interactions with the co-crystallized ligand, i.e., ASP-80, GLY-82, THR-120, GLN-121, TYR-246, and GLY-278. Upon 50 ns-long MD simulations, compound **1**’s stability inside the enzyme’s active site was superior to that of the co-crystallized ligand. (Average RMSD = 1.5 Å and 2.1 Å, respectively).

It is worth noting that despite the poor drug-likeness properties of compound **1**, being a highly hydrophilic compound, it can be absorbed from GIT [70] and also transported via specific carriers across the BBB [71]. The therapeutic potential via the oral route has been proven for similar oligosaccharides, e.g., sodium oligomannate that was authorized by the National Medical Products Administration (NMPA) of China in November 2019 for treating mild to moderate AD [71].

## 4. Discussion

Phytochemical investigation of tamarind pulp *n*-butanol fraction showed it to contain only one new (+)-pinitol glycoside compound **1** (25% *w*/*w*); (+)-pinitol was reported to have a long history as an anti-Alzheimer agent [23,24,72].

Figure 3 shows the study’s findings. The results of the behavioral tests are consistent with earlier findings that showed AlCl_3_-neurointoxicated rats took longer to capture food in the T-maze than control rats, indicating decreased neurocognitive function [41]. Rats using the (+)-pinitol glycoside compound 1 required noticeably less time to find food in the T-maze than those using the AD-induced group, demonstrating improved cognitive ability.

Additionally, AlCl_3_ is reported as a cholinotoxin that provokes functional alterations in the cholinergic, dopaminergic, and noradrenergic neurotransmission. Therefore, it has the propensity to cause impaired cholinergic transmission by affecting the synthesis and release of neurotransmitters [73]. Impaired cholinergic transmission occurs in two ways: First, it occurs either due to a decline in ACh release or decreased choline acetyltransferase activity, which results in the scarcity of ACh. Second, elevated AChE activity further adds to the scarcity of ACh at the synapse by accelerating the decomposition of available ACh; this degradation of ACh is abolished by effective RIVA (AChE-inhibitor) [74]. Moreover, acetyl Co-A synthesis relies on pyruvate formation through energy-dependent glycolysis, which was also found to be altered and therefore justified the deterioration in ACh levels and AChE activity [75]. Furthermore, AlCl_3_-induced oxidative disruption in membrane fluidity/composition can also affect the membrane-bound AChE activity; thus, also corroborating the decreased AChE activity [74]. Our findings demonstrated that administering AlCl_3_ to AD-induced rats resulted in cholinergic impairment as evidenced by a significant increase in cerebral serum AChE activity as compared to the control group. These findings are consistent with Mohamd et al.’s findings from 2011 [76], which showed that AlCl_3_ treatment significantly increased AChE activity in the brain relative to neurologically normal control rats. In comparison to AD rats, (+)-pinitol glycoside compound 1 treatment significantly reduced the brain AChE activity in AD-induced rats (Figure 4).

According to published research, the main causes of mitochondrial dysfunction-induced intracellular damage are believed to be disruptions in antioxidant defense mechanisms and excessive production of reactive oxygen species (ROS) [77]. According to the current findings (Figure 4 and Figure 5), AlCl_3_ induction significantly increased the levels of biomarkers for oxidative damage in brain tissue. This finding is consistent with Aly et al. (2015) [77] declaration that the neurotoxicity associated with AlCl_3_ may be a contributing factor to the elevation in lipid peroxidation. Further reports added a marked elevation in thiobarbituric acid reactive substances in rats brain post-AlCl_3_-induction, which is related to Fe^3+^-carrying protein transfer bonding, hence lowering Fe^2+^ binding and rising free intracellular Fe^2+^ that produces membrane lipids, protein peroxidation, and later membrane destruction, although causing a loss of membrane fluidity, altering membrane potential, elevating permeability of membrane, and disturbing the function of receptors [78]. Additionally, the current study found that the increase in MDA in AD-induced rats was linked to the suppression of antioxidant enzymes, including SOD, GPX, and GSH, which are involved in the elimination of ROS from brain tissue, indicating the pro-oxidant effect of AlCl_3_. Instead, Sumathi et al. (2013) [78] found that exposure to AlCl_3_ causes changes in the enzymatic antioxidant defense system that enhances the breakdown of neuronal lipid. In addition, the data demonstrated a considerable drop in GSH levels in the brain tissue of AlCl_3_-induced rats, which may be explained by a high level of H_2_O_2_-induced cytotoxicity in brain endothelial cells due to glutathione reductase inhibition [77]. Long-term exposure to AlCl_3_ increases lipid peroxidation while depleting and exhausting a number of antioxidant enzymes, which may explain the considerable reduction in brain TAO in AlCl3-induced AD rats [77]. The decrease in axonal mitochondrial transformation, Golgi dysfunction, and a reduction in synaptic vesicles, which lead to the release of oxidative products such as hydroperoxide, carbonyls, and peroxyl nitrites, together with a decrease in antioxidant enzymes and glutathione within the neurons, are additional explanations given by Aly et al. (2018) [77]. The increased concentration of polyunsaturated fatty acids in the brain, which readily interact with developed radicals and enable oxidative destruction in AD-induced rats, also contributes to the high level of Fe that promotes ROS [77]. 

Treatment of rats with (+)-pinitol glycoside compound **1** showed their potent antioxidant activities through increasing the levels of antioxidant defense system GSH, GPX, SOD, and TAC, and reducing MDA in brain tissues and serum (Figure 4 and Figure 5).

The significant increase in serum amyloid-*β* protein and tau protein were able to differentiate between AD-induced rats and neurologically normal controls. This agrees with previous studies by Nayak and Yokel et al. (1999, 2002) [79,80], which demonstrated that AlCl_3_ promotes the accumulation of insoluble A*β* (1–42) protein and A*β* plaque formation. Moreover, the study performed by Pesini et al. (2019) [81] supported the concept that the vascular system is a major player in controlling A*β* levels in the brain; A*β*-plaques appear to be formed if their levels in brain extracellular space surpass the transport capacity of the clearance mechanism across the blood brain barrier (BBB), or if the vascular transport of the peptide was deteriorated and proved that increased blood A*β* levels are an early event that precedes the onset of cognitive decline and increases the risk of developing AD. The current significant increase in serum A*β* peptide levels in untreated AD-induced rats indicated neuronal cytoskeleton disruption induced by AlCl_3_ intoxication led to abnormal accumulation of A*β* peptide in the brain, which is reflected in its high serum level. Consequently, its clearance is considered a primary therapeutic target for managing AD. Furthermore, tau is a neuronal microtubule-associated protein that is primarily found in the axons [82]. In healthy brains, 2-3 tau residues are detected as phosphorylated, while tau is significantly more phosphorylated, with nine phosphates per molecule in AD and other cognitive illnesses [83]. Interestingly, (+)-pinitol glycoside compound **1** showed a significant decrease in A*β* and the ph/T ratio of tau levels when compared to AD rats, reflecting the possible role of polyols in serum A*β* peptide decrement and clearance (Figure 6). Notably, there is no significance difference between prophylactic or therapeutic use of (+)-pinitol glycoside compound **1** in the present study. 

Network pharmacology analysis and docking-based and MD simulation-based investigations indicated the key proteins involved in AD’s pathogenesis, and putatively identified the key proteins that can be targeted by the newly isolated (+)-pinitol glycoside (compound **1**). Further molecular investigation in this regard will be critical to fully understand the mode(s) of action of this compound. 

## 5. Conclusions

In this study, the newly discovered (+)-pinitol glycoside (**1)** from *T. indica* pulp demonstrated remarkable neuroprotective, antiapoptotic, and antiamnesic effects against AlCl_3_-induced cerebral damages and cognitive decline. This action may be related to the compound’s antioxidant and anti-AchE properties. A subsequent network pharmacology study was carried out to analyze the reported molecular targets for human Alzheimer’s disease and determine those that are most important in the pathogenesis of the disease. The potential Alzehimer’s disease-related targets for compound **1** were then identified using an in-depth in silico analysis (including molecular docking, binding free energy calculation (Δ*G*_Binding_), and molecular dynamics simulation). Future in-depth mechanistic research is still required to support the findings of this study, which advocate the use of (+)-pinitol glycoside as a potentially effective medication in the treatment of AD.

## Figures and Tables

**Table 1:** DEPT-Q (400 MHz) and ^1^H (100 MHz) NMR data of compound **1** in CD_3_OD-*d_4_*; carbon multiplicities were determined by the DEPT-Q experiments.

Position	1	
Moiety	*^δ^*_C_	*^δ^*_H_ (*J* in Hz)
(+)-pinitol		
1	84.4, CH	3.28, overlapped
2	73.0, CH	3.92, overlapped
3	73.3, CH	4.55, m
4	72.2, CH	3.71, m
5	74.5, CH	3.70, overlapped
6	71.5, CH	3.81, overlapped
-OCH_3_	60.6	3.63, s
*α*-d-glucopyranosyl		
1′	93.5, CH	5.14, *d* (3.5)
2′	71.4, CH	3.81, overlapped
3′	77.2, CH	4.06, overlapped
4′	69.1, CH	3.81, overlapped
5′	71.6, CH	3.81, overlapped
6′	64.0, CH_2_	3.62, 4.04, m
*β*-d-glucopyranosyl		
1″	97.7, CH	4.51, *d* (8)
2″	73.8, CH	3.62, overlapped
3″	76.3, CH	3.16, t
4″	70.8, CH	3.87, overlapped
5″	77.5, CH	3.35, overlapped
6″	62.4, CH_2_	3.81, 4.04, m
*β*-d-fructofuranosyl		
1‴	65.5, CH_2_	3.50, 3.69, m
2‴	99.0, qC	
3‴	77.6, CH	3.35, overlapped
4‴	71.2, CH	3.31, m
5‴	82.7, CH	3.78, overlapped
6‴	64.3, CH_2_	3.51, 4.04, m

qC, quaternary; CH, methine; CH_2_, methylene; CH_3_, methyl carbons.

## Data Availability

Data are contained within the article.

## Supplementary Materials

The following supporting information can be downloaded at: https://www.mdpi.com/article/10.3390/metabo13060732/s1, Table S1. The information of 82 targets related to Alzheimer’s disease, Table S2. Proteins predicted to be potential targets for compound 1, Figure S1. 1H NMR spectrum of compound 1 measured in CD_3_OD-d_4_ at 400 MHz, Figure S2. DEPT-Q NMR spectrum of compound 1 measured in CD_3_OD-d_4_ at 100 MHz, Figure S3. HSQC spectrum of compound 1 measured in CD_3_OD-d_4_, Figure S4. HMBC spectrum of compound 1 measured in CD_3_OD-d_4_.
