# Supplementary material for: Anti-Alzheimer Potential of a New (+)-Pinitol Glycoside Isolated from Tamarindus indica Pulp: In Vivo and In Silico Evaluations
Source: Metabolites. 2023 Jun 7;13(6):732. doi: 10.3390/metabo13060732 (PMC10304100; doi:10.3390/metabo13060732)
Supplement: Supplementary file 1 [file metabolites-13-00732-s001.zip › metabolites-2382717-supplementary.pdf]

SUPPLEMENTARY DATA

Anti-Alzheimer Potential of a New (+)-Pinitol Glycoside Isolated from *Tamarindus indica* Pulp: *In Vivo* and *In Silico* Evaluations

Esraa M. Mohamed[1], Abeer H. Elmaidomy[2*], Rania Alaaeldin[3], Faisal Alsenani[4], Faisal H. Altemani[5], Naseh A. Algehainy[5], Mohammad A Alanazi[5], Alaa Bagalagel[6], Abdulhamid Althagafi[6], Mahmoud A Elrehany[3], Usama Ramadan Abdelmohsen[7*]

[1]Department of Pharmacognosy, Faculty of Pharmacy, MUST, Giza, 12566, Egypt
[2]Department of Pharmacognosy, Faculty of Pharmacy, Beni-Suef University, Beni-Suef 62511, Egypt
[3]Department of Biochemistry, Faculty of Pharmacy, Deraya University, University Zone, New Minia City 61111, Egypt.
[4]Department of Pharmacognosy, College of Pharmacy, Umm Al-Qura University, Makkah 21955, Saudi Arabia
[5]Department of Medical Laboratory Technology, Faculty of Applied Medical Sciences, University of Tabuk, Tabuk 71491, Saudi Arabia
[6]Department of Pharmacy Practice, Faculty of Pharmacy, King Abdulaziz University, Jeddah 21589, Saudi Arabia
[7]Department of Pharmacognosy, Faculty of Pharmacy, Deraya University, 7 Universities Zone, New Minia 61111, Egypt
Corresponding author: usama.ramadan@mu.edu.eg, abeer011150@pharm.bsu.edu.eg

Table S1. The information of 82 targets related to Alzheimer's disease.

No.	***Target***	***Full name***
1	*APP*	*Beta amyloid A4 (resveratrol and ferulic acid and Chrysin)*
2	*MAOA*	*Monoamine oxidase A (Resveratrol and chrysin)*
3	*MAOB*	*Monoamine oxidase B (Ferulic acid and chrysin)*
4	*ACHE*	*Acetylcholinesterase*
5	*BCHE*	*Butyrylcholinesterase*
6	*PIK3CB*	*PI3-kinase p110-beta subunit*
7	*PIK3CA*	*PI3-kinase p110-alpha subunit*
8	*BACE1*	*Beta-Secretase 1*
9	*BACE2*	*Beta secretase 2*
10	*HTR7*	*Serotonin 7 (5-HT7) receptor*
11	*HTR6*	*Serotonin 6 (5-HT6) receptor*
12	*MAPT*	*Microtubule-associated protein tau*
13	*CDK5*	*Cyclin-dependent kinase 5/CDK5 activator 1*
14	*PTGS2*	*Cyclooxygenase-2 (and resveratrol)*
15	*PSEN1*	*Presenilin 1*
16	*PSEN2*	*Presenilin 2*
17	*APOE*	*Apolipoprotein E*
18	*ABCA7*	*ATP Binding Cassette Subfamily A Member 7*

19	*SNCA*	*Synuclein Alpha*
20	*SORL1*	*Sortilin Related Receptor 1*
21	*ADAM10*	*ADAM Metallopeptidase Domain 10*
22	*A2M*	*Alpha-2-Macroglobulin*
23	*NOS3*	*Nitric Oxide Synthase 3*
24	*PRNP*	*Prion Protein*
25	*TREM2*	*Triggering Receptor Expressed On Myeloid Cells 2*
26	*MT-ND1*	*Mitochondrially Encoded NADH:Ubiquinone Oxidoreductase Core Subunit 1*
27	*HFE*	*Homeostatic Iron Regulator*
28	*PLAU*	*Plasminogen Activator, Urokinase*
29	*SNCB*	*Synuclein Beta*
30	*GRN*	*Granulin Precursor*
31	*MT-ND2*	*Mitochondrially Encoded NADH:Ubiquinone Oxidoreductase Core Subunit 2*
32	*UNC5C*	*Unc-5 Netrin Receptor C*
33	*TOMM40*	*Translocase Of Outer Mitochondrial Membrane 40*
34	*MPO*	*Myeloperoxidase*
35	*GSK3B*	*Glycogen Synthase Kinase 3 Beta*
36	*COMT*	*Catechol-O-Methyltransferase*
37	*PRKN*	*Parkin RBR E3 Ubiquitin Protein Ligase*
38	*CLU*	*Clusterin*
39	*HTR2A*	*5-Hydroxytryptamine Receptor 2A*
40	*LRP1*	*LDL Receptor Related Protein 1*
41	*Serpin Family A Member 3*<br>*MIR29A*	*Serpin Family A Member 3*<br>*MicroRNA 29a*
42	*DRD3*	*Dopamine Receptor D3*
43	*MIR29B1*	*MicroRNA 29b-1*
44	*IDE*	*Insulin Degrading Enzyme*
45	*MIR107*	*MicroRNA 107*
46	*MIR146A*	*MicroRNA 146a*
47	*CHAT*	*Choline O-Acetyltransferase*
48	*CTSD*	*Cathepsin D*

49	*TNF*	*Tumor Necrosis Factor*
50	*APBB1*	*Amyloid Beta Precursor Protein Binding Family B Member 1*
51	*BDNF*	*Brain Derived Neurotrophic Factor*
52	*NCSTN*	*Nicastrin*
53	*PICALM*	*Phosphatidylinositol Binding Clathrin Assembly Protein*
54	*HSD17B10*	*Hydroxysteroid 17-Beta Dehydrogenase 10*
55	*CDK5R1*	*Cyclin Dependent Kinase 5 Regulatory Subunit 1*
56	*COL25A1*	*Collagen Type XXV Alpha 1 Chain*
57	*ACE*	*Angiotensin I Converting Enzyme*
58	*PLD3*	*Phospholipase D Family Member 3*
59	*PSENEN*	*Presenilin Enhancer, Gamma-Secretase Subunit*
60	*CASP3*	*Caspase 3*
61	*CR1*	*Complement C3b/C4b Receptor 1*
62	*IL1B*	*Interleukin 1 Beta*
63	*MME*	*Membrane Metalloendopeptidase*
64	*CALHM1*	*Calcium Homeostasis Modulator 1*
65	*IL1A*	*Interleukin 1 Alpha*
66	*TF*	*Transferrin*
67	*GAPDH*	*Glyceraldehyde-3-Phosphate Dehydrogenase*
68	*SOD1*	*Superoxide Dismutase 1*
69	*IL6*	*Interleukin 6*
70	*PTGS2*	*Prostaglandin-Endoperoxide Synthase 2*
71	*VLDLR*	*Very Low Density Lipoprotein Receptor*
72	*MAPK1*	*Mitogen-Activated Protein Kinase 1*
73	*DHCR24*	*24-Dehydrocholesterol Reductase*
74	*CAPN1*	*Calpain 1*
75	*VCP*	*Valosin Containing Protein*

76	*APLP2*	*Amyloid Beta Precursor Like Protein 2*
77	*CHRNA7*	*Cholinergic Receptor Nicotinic Alpha 7 Subunit*
78	*NGF*	*Nerve Growth Factor*
79	*CLSTN1*	*Calsyntenin 1*
80	*OLR1*	*Oxidized Low Density Lipoprotein Receptor 1*
81	*INS*	*Insulin*
82	*NOS1*	*Nitric Oxide Synthase 1*

Table S2. Proteins predicted to be potential targets for compound1

No.	***Target***	***Full name***	***Uniport ID***	***PharmMapper (Fit score score)***	***Vina score kcal/mol***	***$\Delta G_{binding}$ kcal/mol***
1	*APP*	*Beta amyloid A4*	*P05067*	8.3	-4.9	-4.1
2	*MAOA*	*Monoamine oxidase A*	*P21397*	*8.4*	*-5.5*	*-4.8*
3	*PIK3CB*	*PI3-kinase p110-beta subunit*	*P42338*	8.5	-7.1	-5.4
4	*PIK3CA*	*PI3-kinase p110-alpha subunit*	*P42336*	8.3	-4.2	-4.5
5	*MAOB*	*Monoamine oxidase B*	*P27338*	*8.2*	*-7.6*	*-5.1*
6	***BACE1****	***Beta secretase 1***	***P56817***	***8.7***	***-8.1***	***-7.8***
7	*HTR7*	*Serotonin 7 (5-HT7) receptor*	*P34969*	8.1	-5.3	-4.2
8	*HTR6*	*Serotonin 6 (5-HT6) receptor*	*P50406*	7.6	-5.2	-5.8
9	***ACHE****	***Acetylcholinesterase***	***P22303***	**7.4**	***-7.7***	***-7.1***
10	*BCHE*	*Butyrylcholinesterase*	*P06276*	*8.5*	*-6.3*	*-5.6*
11	*MAPT*	*Microtubule-associated protein tau*	*P10636*	7.2	-6.7	-5.1

*The best scoring protein targets

Molecular Docking

Docking experiments were performed using Auto Dock Vina software according the previously reported protocol[1]. The binding site in each docked protein was determined according to the co-crystalized ligand. To account for these binding sites' flexibility, we used their MDS-derived conformers sampled every 10 ns for docking experiments (i.e., ensemble docking). Subsequently, the retrieved top hits were ranked

according to their binding energies. The generated docking poses were visualized and analyzed using Pymol softwar [2].

Molecular Dynamic Simulation

MD simulations were performed by Desmond v. 2.2 [3] the MDS machine of Maestro software [4] using the OPLS3 forcefield. The protein-ligand systems were built via System Builder option, where it was embedded in an orthorhombic box of TIP3P waters together with 0.15 M Na^{+} and Cl^{-} ions with 20 A° solvent buffer from the molecular surface of the centrally placedreceptor. Afterwards, the prepared system was energy minimized and equilibrated for 10 ns. Desmond software automatically parameterizes inputted ligands during the system building step according OPLS force field. For simulations performed by NAMD, the parameters and topologies of the compounds were calculated either using Charmm27 force field by the online software Ligand Reader & Modeler (http://www.charmm-gui.org/?doc=input/ligandrm) [5] or by the VMD plugin Force Field Toolkit (ffTK) [6]. Afterward, the generated parameters and topology files were loaded to VMD so that it can readily read the protein-ligand complexes without errors and then conduct the simulation step. Simulations were run for 50 ns at 310 K in the NPT ensemble with the Nose-Hoover thermostat and Martyna-Tobias-Klein barostat using an anisotropic coupling. We used the best binding poses for each compound-protein complex as starting systems to investigate their binding stability and mode of interactions.

Binding free energy calculations (ΔG) were performed using the free energy perturbation (FEP) method. We first prepared the input files and script NAMD by the online-based software CHARMM-GUI Free Energy Calculator (http://www.charmm-gui.org/input/fec). Afterwards, these inputs were loaded to NAMD for simulations, where the equilibration was performed in the NPT ensemble at 300 K and 1 atm (1.01325 bar) with Langevin piston pressure (for "Complex" and "Ligand") in the presence of TIP3P water model. 10 ns FEP simulations were performed fro each compound, and the last 5 ns of the free enegry values was measured for the final free energy values [7].

Networks Construction

We constructed two networks (Figures2 and 3): (i) compound-protein interaction (CPI) network depending on the results of prediction. In this network we constructed connections between the metabolites that showed drug-like properties and predicted to pass the BBB, and target proteins relevant to AD; (ii) protein-protein interaction (PPI) network that showed the interactions between proteins relevant to AD. The proteins that were found to be potential targets for the select metabolites were submitted to the STRING database (https://string-db.org/) [8] for protein–protein interaction (PPI) analysis, and "Homosapiens" was selected as the search species., the lowest interaction score was set to 0.4, and the rest of the parameters were set to the default setting to obtain the PPI network .All of the above networks were constructed and summarized in two figures (Figure 2 and 3) using Cytoscape 3.8.2 (https://www.cytoscape.org/) [9], which is a software package for visualizing and analyzing networks.

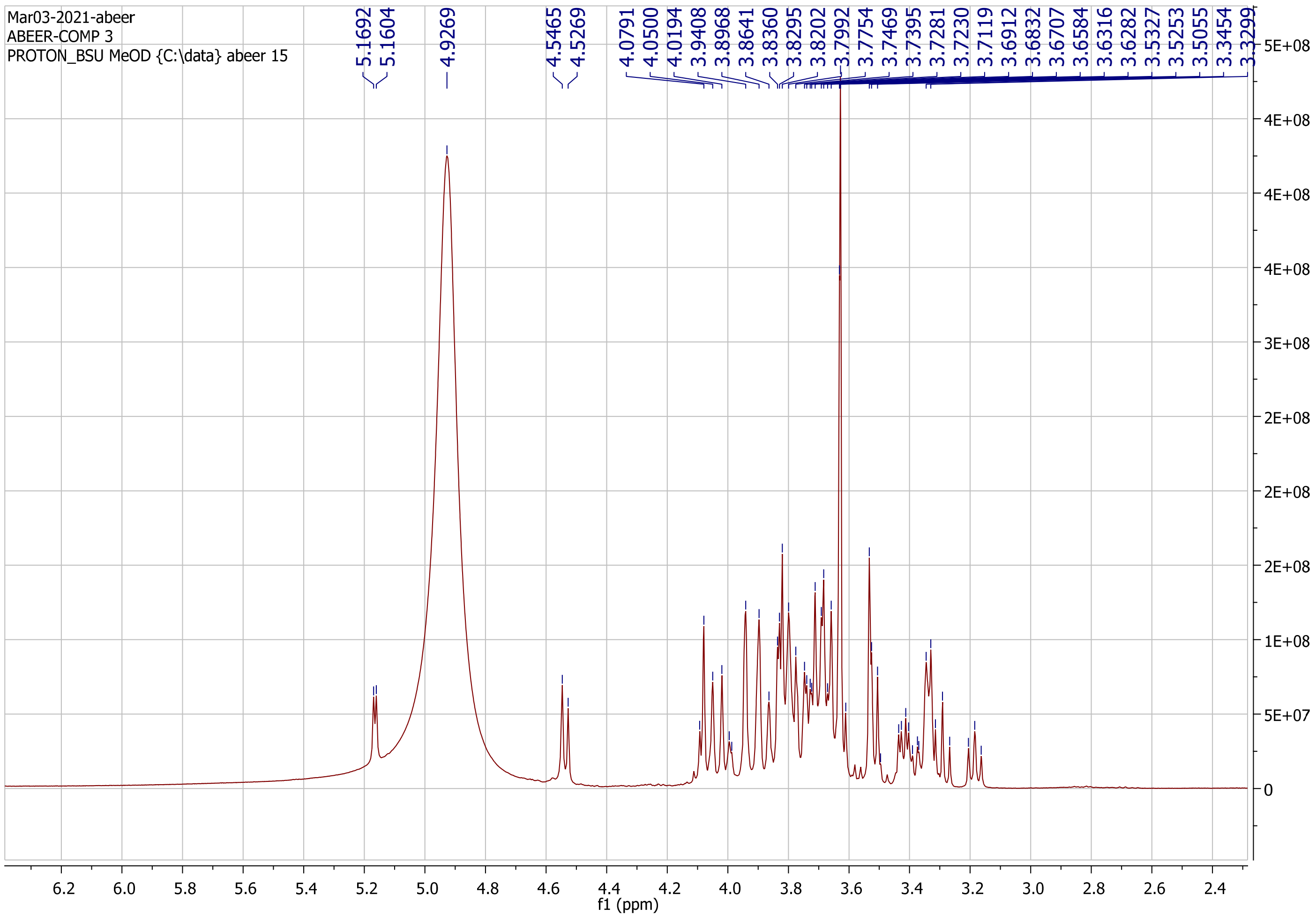

Figure S1. 1H NMR spectrum of compound **1** measured in CD_3OD-d_4 at 400 MHz

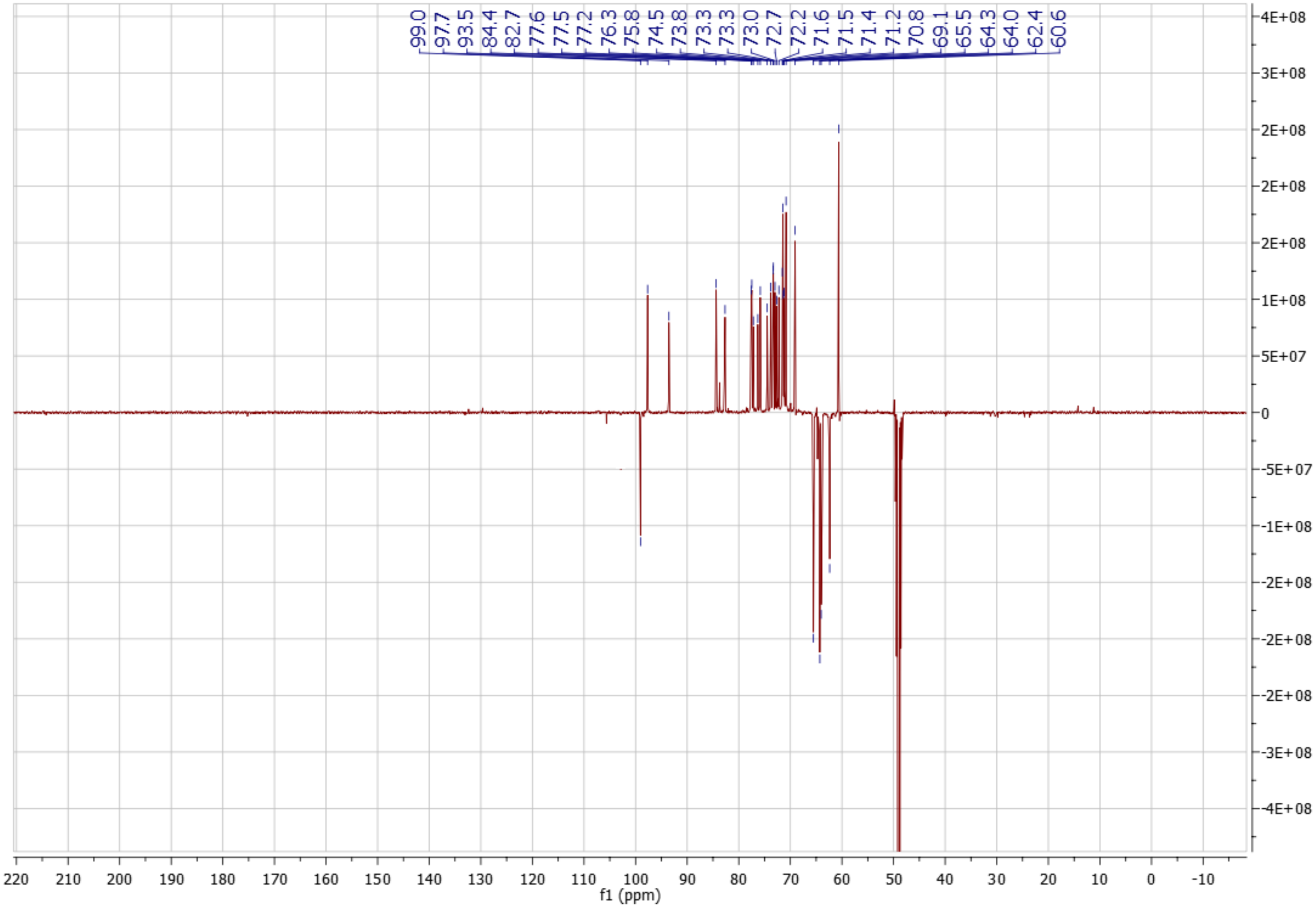

Figure S2. DEPT-Q NMR spectrum of compound **1** measured in CD_3OD-d_4 at 100 MHz

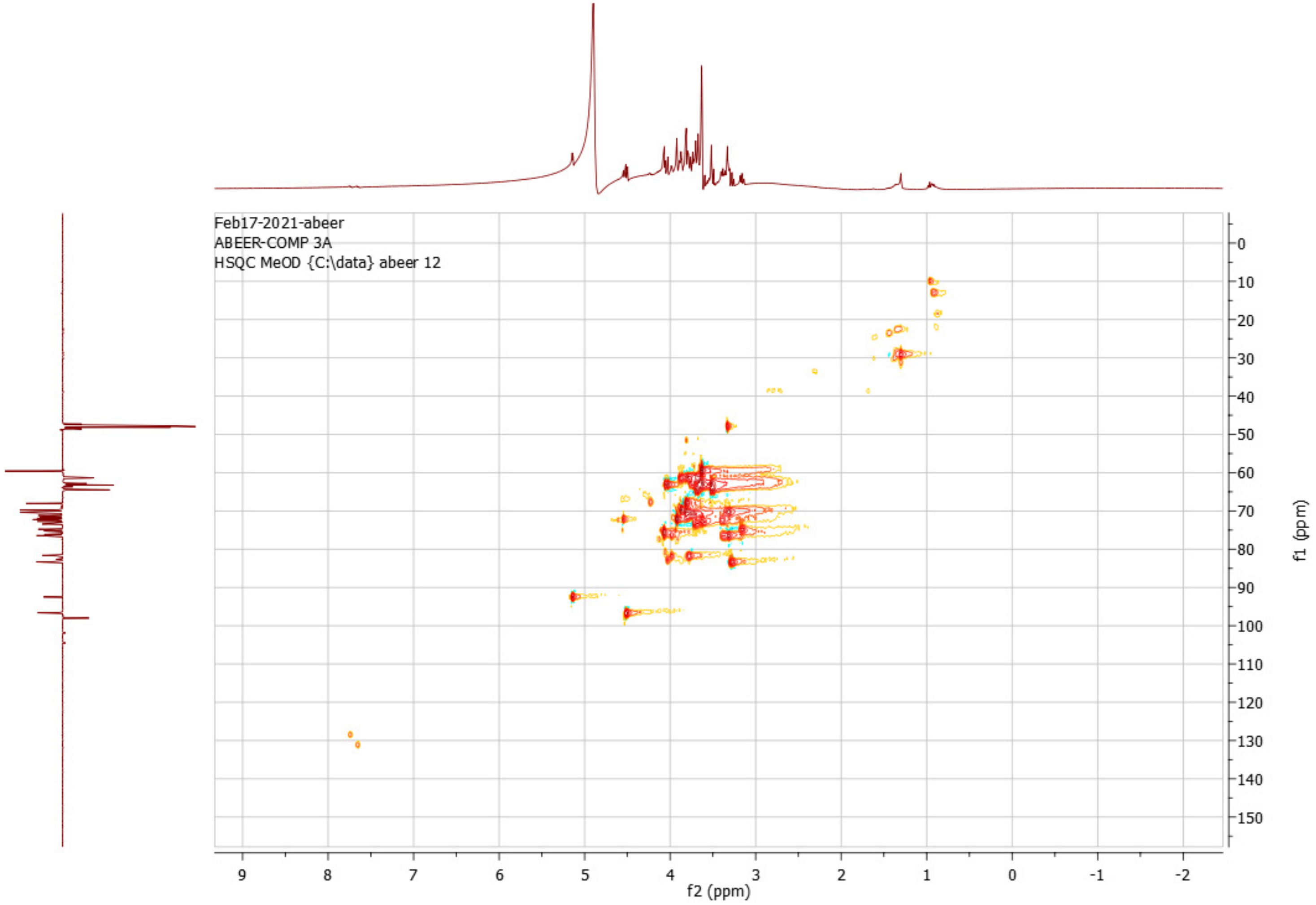

Figure S3. HSQC spectrum of compound **1** measured in CD_3OD-d_4

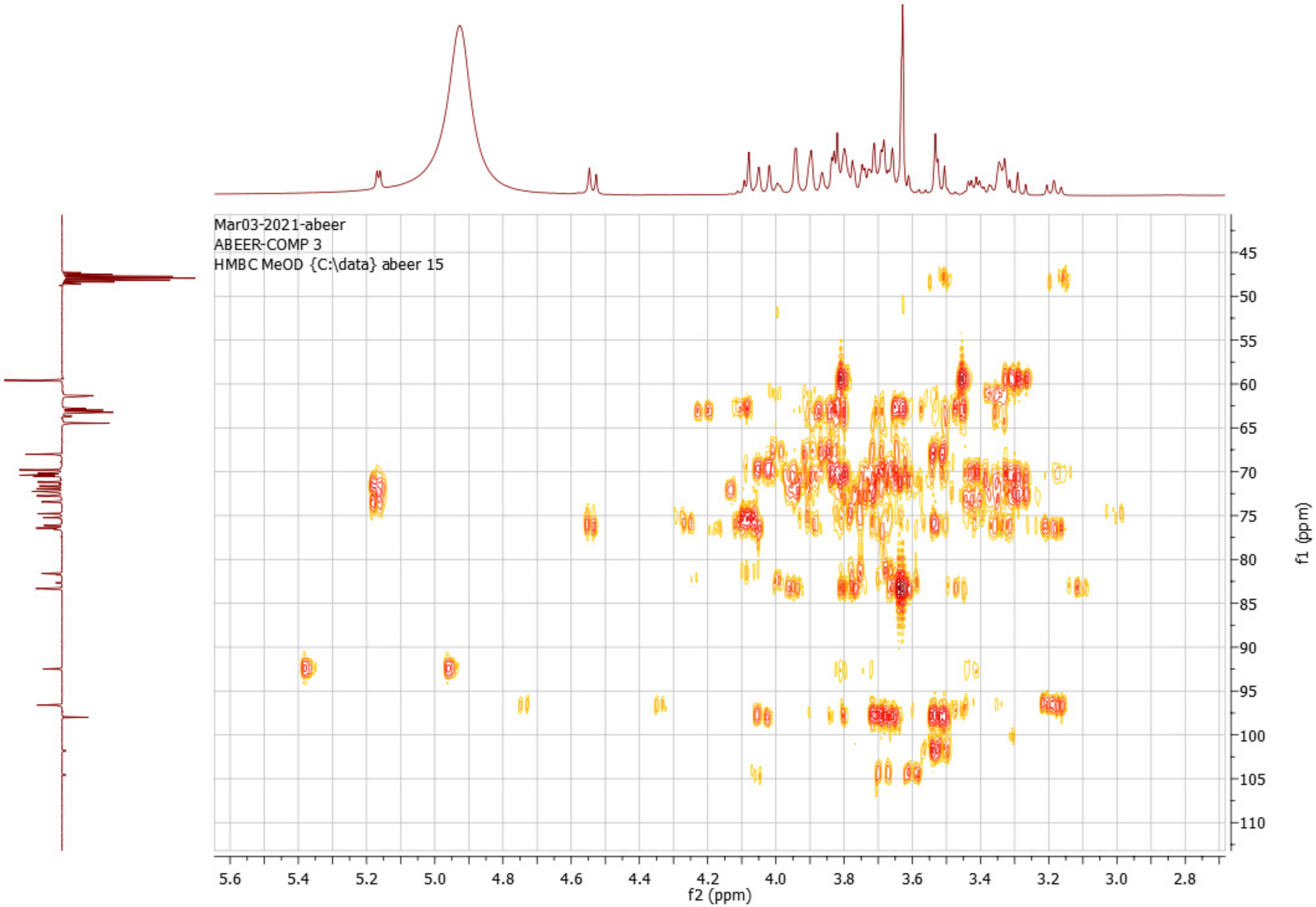

Figure S4. HMBC spectrum of compound **1** measured in CD_3OD-d_4

References

1. Huey, R., Morris, G. M., & Forli, S. (2012). Using AutoDock 4 and AutoDock vina with AutoDockTools: a tutorial. *The Scripps Research Institute Molecular Graphics Laboratory*, *10550*(92037), 1000.

2. Yuan, S., Chan, H. S., & Hu, Z. (2017). Using PyMOL as a platform for computational drug design. *Wiley Interdisciplinary Reviews: Computational Molecular Science*, *7*(2), e1298.

3. Bowers, K. J., Chow, E., Xu, H., Dror, R. O., Eastwood, M. P., Gregersen, B. A., ... & Shaw, D. E. (2006, November). Scalable algorithms for molecular dynamics simulations on commodity clusters. In *Proceedings of the 2006 ACM/IEEE Conference on Supercomputing* (pp. 84-es).

4. Galande, A. K., & Rohane, S. H. (2021). Insilico Molecular docking analysis in Maestro Software.

5. Jo, S., Kim, T., Iyer, V. G., & Im, W. (2008). CHARMM-GUI: a web-based graphical user interface for CHARMM. *Journal of computational chemistry*, *29*(11), 1859-1865.

6. Humphrey, W., Dalke, A., & Schulten, K. (1996). VMD: visual molecular dynamics. *Journal of molecular graphics*, *14*(1), 33-38.

7. Gapsys, V., Michielssens, S., Peters, J. H., de Groot, B. L., & Leonov, H. (2015). Calculation of binding free energies. *Molecular Modeling of Proteins*, 173-209.

8. Mering, C. V., Huynen, M., Jaeggi, D., Schmidt, S., Bork, P., & Snel, B. (2003). STRING: a database of predicted functional associations between proteins. *Nucleic acids research*, *31*(1), 258-261.

9. Saito, R., Smoot, M. E., Ono, K., Ruscheinski, J., Wang, P. L., Lotia, S., ... & Ideker, T. (2012). A travel guide to Cytoscape plugins. *Nature methods*, *9*(11), 1069-1076.
